# Management of Diabetic Foot in an Indian Clinical Setup: An Opinion Survey

**DOI:** 10.7759/cureus.8636

**Published:** 2020-06-15

**Authors:** Ashok Das, Sharad Pendsey, Mahesh Abhyankar, Rohit Malabade

**Affiliations:** 1 Medicine and Endocrinology, Pondicherry Institute of Medical Sciences, Pondicherry, IND; 2 Diabetology, Step-by-Step Foot Care Project, Nagpur, IND; 3 Scientific Services, USV Private Limited, Mumbai, IND

**Keywords:** diabetic foot, diabetes mellitus, diabetic foot education program, management of diabetic foot

## Abstract

Objective

The goal of this study was to evaluate foot-care practices by physicians throughout India who had participated in the Diabetic Foot Education Program (DFEP).

Methods

A structured questionnaire was administered to physicians throughout India, and their responses were analyzed descriptively.

Results

A total of 377 doctors responded to the DFEP opinion survey, including 261 doctors who belonged to independent diabetic foot clinics. Of these doctors, 44.4% reported managing fewer than five diabetic foot patients per week and 42.8% reported managing 5-10 patients per week. Most of these patients had non-ischemic foot, followed by those with ischemic and Charcot foot. About 58% of these physicians reported performing comprehensive clinical examinations and providing optimal preventive and therapeutic care in the treatment of diabetic foot, whereas only 25.7% reported performing only callus removal and changing dressings. Basic instruments to manage diabetic foot included the monofilament, tuning fork, biothesiometer, handheld Doppler, and pedometer, which were used by 76%, 75.5%, 59.5%, 27.7%, and 12.8% of doctors, respectively. The most common comorbidities were neuropathy, reported by 333 doctors, followed by peripheral vascular disease, reported by 297 doctors. Tools for diabetic foot education included posters in the clinic, used by 75% of doctors; pamphlets, used by 56.2%; videos, used by 45.2%; and diabetic foot applications, used by 36.7% of doctors.

Conclusions

There is a need to promote diabetic foot awareness and implement foot-care strategies to prevent diabetic foot and effectively manage this condition. Diabetic foot education programs will encourage clinicians to effectively use diagnostic tools for assessment and management of diabetic foot and to establish independent diabetic foot clinics.

## Introduction

Globally, an estimated 463 million adults are living with diabetes; India, with 77 million patients, has the second-highest number of patients after China [1]. Diabetic foot disease represents a real challenge to national health systems and healthcare providers in general [2]. The lifetime risk of a person with diabetes having a foot ulcer has been reported to be as high as 25%, with foot ulcers being the most frequent reason for hospitalization of patients with diabetes (about 30%) [3]. Moreover, treating diabetic foot ulcers is costly, accounting for 20% of total healthcare costs for diabetes, which is more compared to the cost for any other diabetic complication [3]. In India, the numbers of diabetic foot patients are increasing in both urban and rural settings, with 85% of amputations preceded by foot ulcers. Almost 75% of these amputations are performed on neuropathic feet with secondary infection, which is potentially preventable. In India, neuropathic lesions account for 80% of foot ulcers, with neuroischemic making up the remaining 20% [4]. The prevalence of the peripheral arterial disease is 3.2% in diabetic patients aged <50 years, but it increases to 33% in those aged >80 years, with the increase being associated with both age and the duration of diabetes [5]. In India, approximately 100,000 legs are amputated every year, and the numbers are increasing [3].

Diabetes foot management is based on comprehensive patient and wound assessment. Arterial inflow and infection control should be ensured, the degrees of sensory neuropathy and deformity evaluated, and trauma (footwear) and pressure (offloading) should be reduced [6]. Diabetic foot management should focus on both prevention and treatment. Patients should be educated about proper foot hygiene, skin and nail care, proper footwear, and appropriate foot care administered by qualified professionals, all of which can reduce injuries that may lead to foot ulceration [7]. Effective diabetic foot care should involve an interdisciplinary approach, for which a trained team is indispensable [8]. The Diabetic Foot Education Program (DFEP) is a pan-India program designed to increase physician knowledge about the prevention of diabetic foot and its complications, management of diabetic foot (debridement and offloading), foot-care education, and methods to improve the quality of healthcare in diabetic foot patients.

The DFEP India was launched under the auspices of the International Working Group on the Diabetic Foot, the Southern Arizona Limb Salvage Alliance, and the Indian College of Physicians. The program focuses on early detection, identification of at-risk foot, and preventive care. A national opinion survey was undertaken to gather insights from doctors who participated in DFEP India regarding their strategies to prevent and manage diabetic foot. The objective of this survey was to evaluate healthcare practices used by doctors in the diagnosis and initial management of diabetic foot disease in their routine clinical practice.

## Materials and methods

The contents of the diabetic foot questionnaire survey (Table 1) were developed by an expert panel of the DFEP. The questionnaire focused on day-to-day clinical experience, techniques used for diagnosis, approaches to patient management, types of patients referred, and future prospects of the foot education program. The questionnaires were sent to healthcare professionals throughout India and were supposed to be filled out within the stipulated time period. Data were recorded and descriptive analyses performed. Responses were calculated as absolute frequencies and reported as overall percentages.

**Table 1:** Questionnaire on diabetic foot provided to healthcare professionals

Are you running an independent diabetic foot clinic?
Which of the techniques do you use in the management of diabetic foot?
Which approach do you use in the management of diabetic foot?
Which patient activities do you use for diabetic foot education?
What outreach program can be started in your diabetic foot-care clinic?
How many diabetic foot cases do you see in a week?
What percentage of foot cases do you see are non-ischemic, ischemic, or Charcot foot?
What is the clinical cure rate for diabetic foot ulcers?
How many diabetic foot ulcer patients do you refer to specialists per week?
Which are the specialists to whom you refer these diabetic foot cases?
What are the comorbidities you encounter in diabetic foot patients?

## Results

A total of 377 doctors throughout India responded to the DFEP opinion survey, including 261 doctors who belonged to independent diabetic foot clinics.

In-clinic diabetic foot management

**Figure 1:**
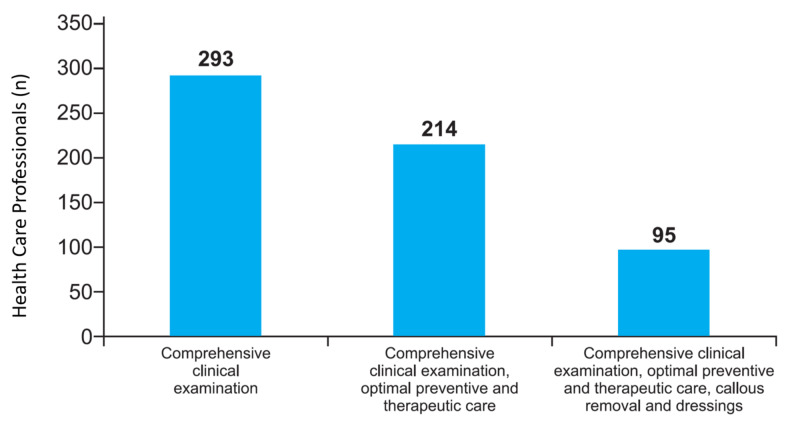
Tools used in the management of diabetic foot

Assessments of in-clinic management of diabetic foot found that approximately 79.4% of doctors performed comprehensive clinical examinations, 58% utilized optimal preventive and therapeutic care strategies along with comprehensive clinical examination, and only 25.7% performed comprehensive clinical examination, utilized optimal preventive and therapeutic care methods, removed calluses, and changed dressings (Figure 1).

Instruments used for the detection of diabetic foot and diabetic foot education

Basic instruments used for the detection and management of diabetic foot included the monofilament, used by 67% of doctors; the tuning fork, used by 75.5%; the biothesiometer, used by 59.5%; handheld Doppler, used by27.7%; and the pedometer, used by 12.8%. Tools for diabetic foot education included posters in the clinic, used by 75% of doctors; pamphlets, used by 56.2%; videos, used by 45.2%; and diabetic foot applications, used by 36.7% (Table 2).

**Table 2:** Patient education tools for diabetic foot

Instruments	Number of doctors (%)
Posters	274 (75%)
Pamphlets	205 (56.2%)
Videos	165(45.2%)
Diabetic foot apps	134 (36.7%)

Diabetic foot clinic outreach programs

Physicians in this survey reported that outreach programs feasible for use in diabetic foot clinics could include methods of health education about diabetic foot, such as pamphlets, videos, and audiovisual aids. Awareness camps, foot-care education programs, and diabetic foot prevention counseling programs involving interactions between doctors and patients were also reported useful, as were discussions between doctors and patients on customized footwear for patients with diabetic foot ulcers. Other outreach programs feasible for diabetic foot clinics should include training of staff members and other assistants, providing information to patients via electronic media, mass education, mobile foot education clinics, Wi-Fi portals for foot care, diabetic foot educators, and daycare for children of patients undergoing diabetic foot surgery.

Management of diabetic foot patients

Among the doctors in this survey, 44.4% reported managing fewer than five patients per week with diabetic foot, 42.8% reported managing 5-10 patients per week, and 8.8% of doctors reported managing 10-25 patients per week. Only 4.0% of the doctors surveyed reported managing >25 patients per week with diabetic foot. Most patients with diabetic foot who visited clinics had non-ischemic foot, followed by those with ischemic and Charcot foot.

Cure rates in diabetic foot ulcers patients

**Figure 2:**
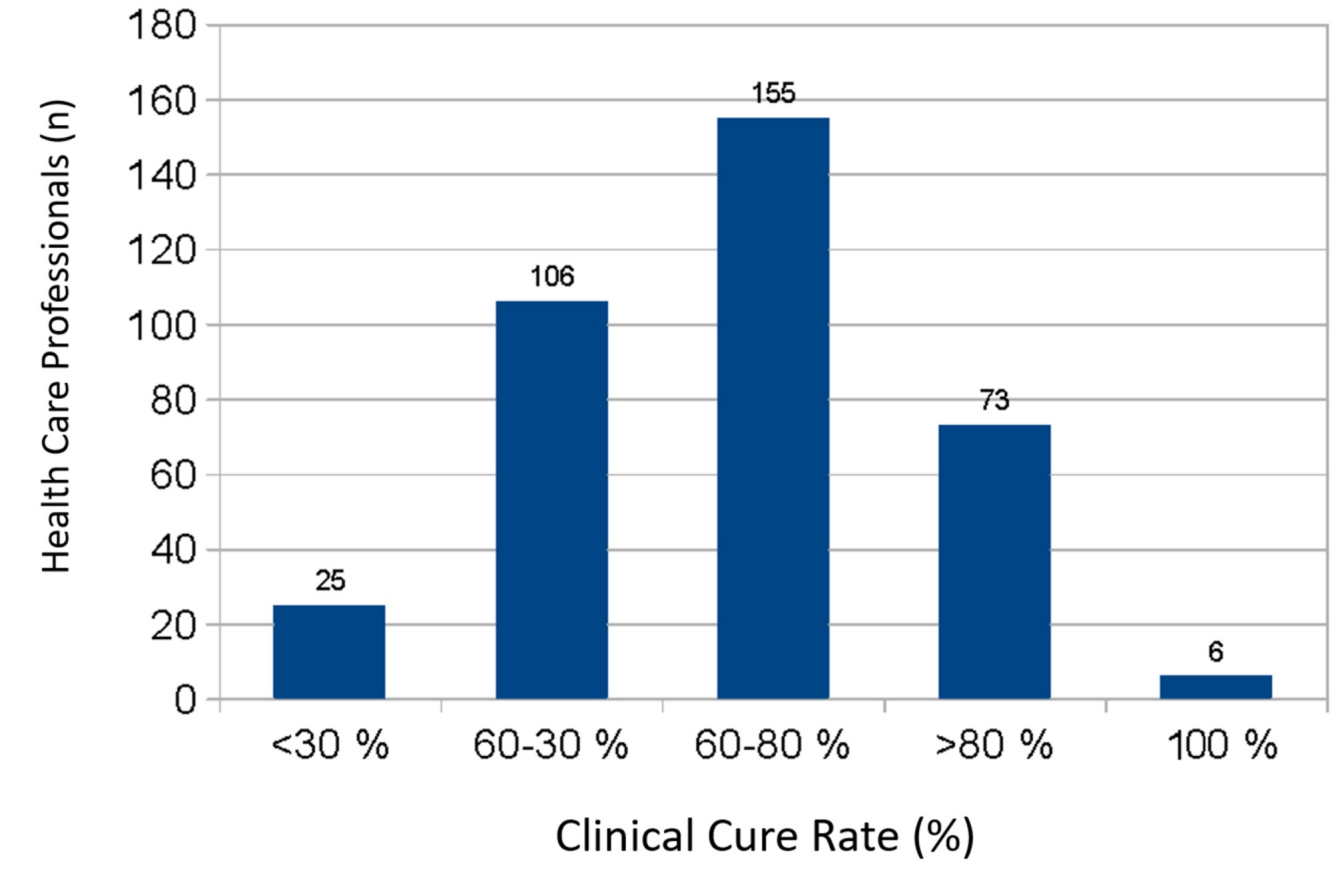
Clinical cure rates in patients with diabetic foot ulcers as reported by healthcare professionals

Approximately 20% of doctors reported clinical cure rates of >80%; 42.5% reported clinical cure rates of 60-80%, and 29.0% reported clinical cure rates of 30-60%. Only 1.6% of doctors reported a cure rate of 100% (Figure 2).

Comorbidities encountered with diabetic foot

**Figure 3:**
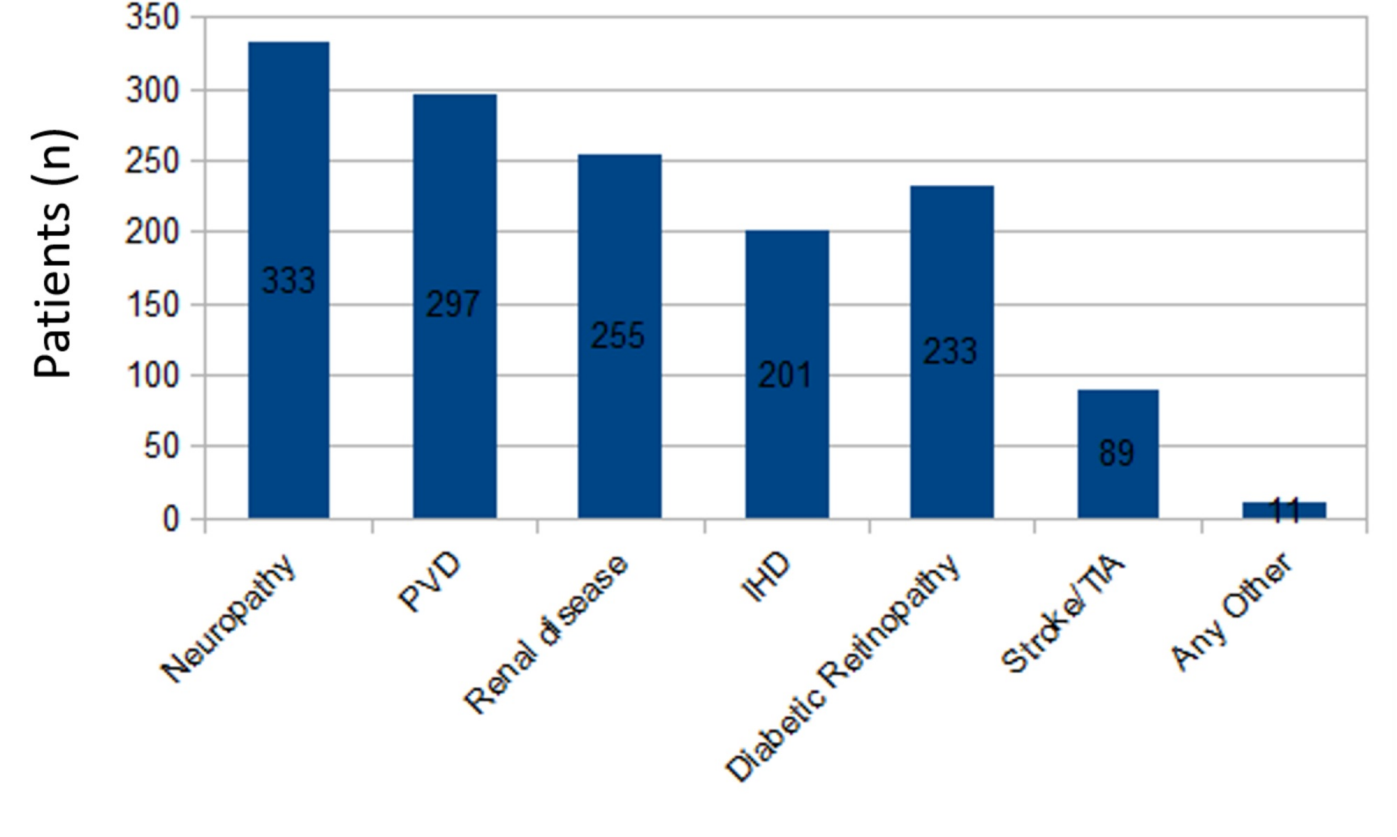
Comorbidities in patients with diabetic foot PVD: peripheral vascular disease; IHD: ischemic heart disease; TIA: transient ischemic attack

Most doctors reported comorbidities in their diabetic foot patients. Of the 377 doctors, 333 (88.3%) reported neuropathy, 297 (78.8%) reported peripheral vascular disease, 255 (67.6%) reported renal disease, and 233 (61.8%) reported diabetic retinopathy in their patients with diabetic foot (Figure 3).

Referral to specialists

The survey showed that 87.8% of physicians reported referring up to five diabetic ulcer patients per week to specialists, whereas 7.2% of doctors referred 6-10 patients per week. Of these doctors, 59.1% referred these patients to general surgeons, 44.2% to vascular surgeons, 29.0% to plastic surgeons, and 27.9% to orthopedic surgeons (Table 3).

**Table 3:** Specialists to whom diabetic foot patients are referred

Specialists	Number of doctors (%)
Surgeons	218 (59.1%)
Vascular surgeons	163 (44.2%)
Plastic surgeons	107 (29.0%)
Orthopedic surgeons	103 (27.9%)

## Discussion

The prevalence of diabetes mellitus in India stands at 8.8% (among people between 20-79 years of age) [1]. The diabetic foot has become one of the most common and serious complications of diabetes mellitus and is a frequent cause of hospitalization and disability [9]. Diabetic foot ulcers were found in 4.54% of patients newly diagnosed with type 2 diabetes mellitus in India; of these, 46.1% had neuropathic, 19.7% had ischemic, and 34.2% had neuroischemic foot ulcers [10].

The present survey found that 261 (69.2%) of the 377 doctors who participated in the DFEP opinion survey practiced at independent diabetic foot clinics. The most common comorbid condition in diabetic foot patients was neuropathy, which puts patients at increased risk of mechanical and thermal trauma without being aware of the injury [11]. Non-ischemic diabetic foot disease was the most commonly managed type of foot disease in this survey. Most of the patients with diabetic foot had comorbid conditions, such as neuropathy, peripheral vascular disease, renal disease, ischemic heart disease, diabetic retinopathy, and stroke/transient ischemic attack, and all of these are conditions that increase mortality rates in patients with diabetes and diabetic foot ulcers [12]. The present survey also found that diabetic foot programs could raise awareness among doctors to provide advice to diabetic patients on foot care at home and work, to maintain good hygiene, and to alleviate foot complications. Ideal neuropathic foot ulcer management is based on comprehensive patient and wound assessment, which, along with debridement and reduced plantar pressure, promotes wound healing [13]. However, only 25.7% of doctors in this survey used all these services, which included clinical examination, optimal preventive and therapeutic care, callus removal, and change of dressing in the management of patients with diabetic foot. Hence, standardized foot care and access to foot-care specialist services across the country are needed.

Early assessment of peripheral sensory neuropathy and peripheral circulation, using efficient, inexpensive, and non-invasive measurement tools, is important [14]. The survey found that around 76% of doctors use various tools to screen for and diagnose diabetic foot. Also, most doctors provided diabetic foot education with in-clinic posters, followed by pamphlets and videos in patient waiting areas. Additionally, 87.8% of doctors reported that they refer up to five diabetic foot ulcer patients per week to specialists [15]. It is necessary to propose a framework and medical policies that support specific foot-care practices that can be provided by all healthcare professionals managing patients with diabetes who are at risk of developing foot complications [16]. The DFEP has been found successful in improving diabetic foot management and care in India by developing reference documents and medical measures for use by all healthcare providers involved in diabetic foot care.

## Conclusions

Approach to diabetic foot management varies in primary and secondary healthcare. However, physicians are effectively using diagnostic tools to assess the nature and severity of diabetic foot. Physicians who were trained in the management of diabetic foot have reportedly been able to manage their patients better initially, with a good success rate. A unified approach for foot-care management may be achieved by diabetes foot education programs and the provision of national foot-care guidelines. Diabetes foot education programs should be implemented nationwide throughout India to create awareness regarding the prevention of the condition and for improving the management of diabetic foot patients. This may reduce foot amputation rates and decrease the burden of diabetic foot throughout India.
